# Prevalence of breast and ovarian cancer subtypes in Hispanic populations from Puerto Rico

**DOI:** 10.1186/s12885-018-5077-z

**Published:** 2018-11-27

**Authors:** Ariel Rodriguez-Velazquez, Rosa Velez, Jean Carlo Lafontaine, Claudia B. Colon-Echevarria, Rocio D. Lamboy-Caraballo, Ingrid Ramirez, Adalberto Mendoza, Patricia Casbas-Hernandez, Guillermo N. Armaiz-Pena

**Affiliations:** 1 Department of Basic Sciences, Division of Biochemistry, School of Medicine, Ponce Health Sciences University, Ponce, PR USA; 2 Southern Pathology Services Inc, Ponce, PR USA; 3 Department of Basic Sciences, Division of Pharmacology, School of Medicine, Ponce Health Sciences University, Ponce, PR USA; 4 Department of Pathology, School of Medicine, Ponce Health Sciences University, Ponce, PR USA; 5 Department of Obstetrics and Gynecology, School of Medicine, Ponce Health Sciences University, Ponce, PR USA; 6 Division of Cancer Biology, Ponce Research Institute, Ponce, PR USA

**Keywords:** Breast cancer, Ovarian cancer, Puerto Rico, Health disparities, Hispanics, Latinos

## Abstract

**Background:**

Previous epidemiological studies aimed at describing characteristics of breast (BC) and ovarian cancer (OC) patients tend to examine Hispanic populations using a mix of individuals that come from ethnically different Hispanic backgrounds. Since most USA cancer statistics do not include cancer data from Puerto Rico (PR), there is a lack of historical and descriptive data analysis for Hispanic women in the island that suffer from these diseases. Therefore, the aim of our study is to provide a comprehensive clinicopathological characterization of BC and OC cases in PR.

**Methods:**

Our study consisted of a longitudinal retrospective review of archived pathology reports at Southern Pathology Services (SPS), which mostly serves southwestern PR, from years 2000–2015. After filtering SPS records with pre-established criteria, tumor samples from 3451 BC and 170 OC cases were used for descriptive statistics and analysis using R program.

**Results:**

In our cohort, the mean age of diagnosis for BC was 60.5 years and 60.3 years for OC. Available data for subtype characterization from BC cases, exhibited an expected subtype distribution that remained stable over time (Luminal A = 68.8%, Luminal B = 9.7%, HER-2 = 6.1% and Triple negative = 15.4%). Additionally, tumor grades distribution varied within different BC subtypes in which the majority of Luminal A tumors were G2 and most Triple negative tumors were G3. For OC cases, available subtype and tumor grade information identified serous histology in 64.71% of all cases and G3 as being the most prevalent tumor grade. Pathology reports revealed that 39.42% of all OC cases were described as late stage, while 50.5% as early stage (by pathological staging).

**Conclusion:**

Our data suggests that OC and BC subtypes distribution in Hispanic populations from PR are in-line with national averages. In a significant number of BC cases, subtype could not be determined due to study limitations, health insurance coverage, or other reasons described here and may constitute a health disparity. Altogether, and despite these gaps, this study represents one of the most complete reviews of BC and OC in PR and provides an opportunity to further study this population separate from other US Hispanic populations.

**Electronic supplementary material:**

The online version of this article (10.1186/s12885-018-5077-z) contains supplementary material, which is available to authorized users.

## Background

Breast cancer (BC) is the most common type of cancer in women and the leading cause of death among women in the United States (USA) [1]. In contrast, ovarian cancer (OC) accounts for 3% of all cancers in women but is the leading cause of mortality among gynecological cancers [2]. Cancer heterogeneity can be influenced by multiple factors including race and ethnicity. Although BC is the most common cancer among Hispanic women, they tend to have lower incidence and mortality rates than non-Hispanic whites in the USA [3]. However, Hispanic women are usually diagnosed at advanced stages when compared to non-Hispanic white patients [3, 4]. Hispanics with OC living in the USA are diagnosed at earlier stages and have longer median survival rates than non-Hispanic whites and African-Americans [5]. Many epidemiological studies describing BC and OC patient characteristics, such as SEER, have been conducted with a mix of individuals from different Hispanic backgrounds. Most of these studies have neglected the distinct genetics and environmental exposures in different Hispanic subgroups, which reduce the generalizability of their conclusions to specific Hispanic patients such as Puerto Rican women [6].

Beyond tumor stage and grade, different BC and OC subtypes have been described according to morphological and more recently molecular characteristics of the tumor [7–9]. These subtypes are of crucial importance for patient management and clinical outcomes, [10] especially in BC. Four main subtypes of BC have been established based on immunohistochemistry (IHC) biomarkers: Luminal A (estrogen and/or progesterone-receptor positive, HER-2 negative and have the best prognosis), Luminal B (estrogen and/or progesterone-receptor positive and either HER-2 positive or HER-2 negative), HER-2 overexpressed and Triple negative (which are negative for the three main biomarkers and have the worst prognosis) [11]. In OC, there are no consensus molecular subtypes yet, and tumors are characterized based on histopathologic characteristics into five main subtypes: high-grade serous, endometrioid, clear cell, mucinous, and low-grade serous carcinoma [12]. Contrary to BC, in OC, the tumor stage determines the treatment and follow up for the patient, not the subtype.

The prevalence of BC and OC subtypes in Hispanics populations from Puerto Rico remains unknown. Many USA-wide statistics do not include Puerto Rico [1] in their data and the most recent data available from the Puerto Rico Cancer Registry dates back to 2012. Data from this registry identifies BC and OC as the first and eighth most common cancer among Puerto Rican women, with BC as the primary cause of cancer-related deaths [13]. Additional information such as stage, grade or molecular subtype is not currently available. Even though several studies have shed light on the biology, epidemiology and access to care of BC and OC Puerto Rican patients [14, 15], a current and comprehensive analysis is warranted to determine the state of these diseases among Hispanic women in Puerto Rico.

Given the limited available data on BC and OC in Puerto Rico, our study aims to provide a descriptive analysis of clinicopathological characteristics of BC and OC in Puerto Rican women through a retrospective analysis of pathology reports.

## Methods

### Overview

This study was a longitudinal retrospective review of archived pathology reports at Southern Pathology Services (SPS), located in Ponce, Puerto Rico, from years 2000 to 2015. SPS mostly serves southwestern Puerto Rico and specializes in anatomic, clinical and molecular pathology services. SPS uses the Windopath Laboratory Information System (LIS) version 7.1 to store all electronic data regarding tests and procedures done at their facilities. The study protocol related to this retrospective analysis of de-identified demographical and clinicopathological characteristics of BC and OC was approved by the Institutional Review Board from the Ponce Health Sciences University (IRB approval number: 151207-PC).

### Study population and data extraction

Our study population only included female BC and OC patients, between the ages of 21–89 who had the pathology review of their tumors performed at SPS. The BrioQuery software version 6.6 was used to identify and extract information from patient samples analyzed at SPS with a first primary diagnosis of BC and OC (i.e., recurrent or relapsed cases were excluded) recorded between January 1, 2000 and December 31, 2015. Briefly, data from SPS was filtered using the following pre-established criteria. The first filter applied to the SPS database identified if cases were cancerous; we used keywords such as “carcinoma”, “invasive”, “in situ” and “metastatic” (those cases that did not contain any of these keywords were eliminated from the database). The second filter separated the databases into two, one for BC and one OC; we included the words “breast” and “mammary” for BC cases and “ovary”, “ovaries” and “ovarian” for OC cases (cases that did not contain any of these keywords were eliminated from the database). Once these filters were applied, we revised each individual case to ensure that each case complied with our inclusion criteria (female, BC or OC diagnosis and between the ages of 21–89). To ensure that data from only one diagnostic report per patient was included in the study, multiple reports (issued either in the same or in different years) belonging to the same patient were coded by SPS personnel with the same research ID. Diagnosis from each report belonging to the same patient was reviewed and only data from the initial diagnostic report was conserved but data from subsequent reports (i.e. pathological reports from specimen resections after an initial diagnosis from a biopsy) were missing or not available to the researcher. The final database included demographic and clinicopathological information from tumor samples (*n* = 3451 primary BC; *n* = 170 primary OC) analyzed from 2000 to 2015 at SPS.

### Study variables

All study variables were extracted from de-identified individual pathology reports in the BrioQuery 6.6 database.

#### Demographical variables

From de-identified pathology reports we obtained the following variables: age of the patient when sample was collected (only patients from 21 to 89 years of age were included in the database, patients under or over this age range were excluded by SPS prior to providing the database). This is a self-reported variable obtained from patient at the moment the physician requests the sample to be analyzed. All of our participants were female (all male participants were excluded by SPS prior to providing the database). The geographical variable (categorized as North, South, East, West and Metropolitan Area) was obtained by cross-referencing health care provider address in BrioQuery 6.6 with municipality and then classifying municipalities by geographical location on the island following previous classification systems [16].

#### Clinical and pathological variable

Pathological data included: histological classification, tumor grade, pathological stage, primary tumor size, and biomarker status. Primary tumor site was defined as the origin of the primary tumor; a tumor site was considered to be primary breast or ovarian if the pathology report stated that the origin of the cancer was either breast or ovary. Cases were also considered to be primary breast or ovarian if the pathology report did not state origin of tumor but the only site with cancerous cells was the breast or ovary. If cases had multiple sites of cancer and no primary site was stated, the case was excluded from the analysis. OC histology was determined by a staff pathologist upon examination of the hematoxylin and eosin (H&E) stained slides. OC type was classified as follows: clear cell/squamous, endometrioid, mucinous and serous [9]. A group of cases with an uncommon histological classification (i.e granulosa cell, transitional cell carcinoma, dygeminoma and mullerian adenocarcinoma, *n* = 11) were classified as other. Tumor grade was defined according to the AJCC **(**American Joint Committee on Cancer Cancer Staging Manual 2017) [20]: G1 (Well differentiated, low grade), G2 (Moderately differentiated, intermediate grade), G3 (Poorly differentiated, high grade). The pathological stage of the tumor was determined by pathologists using the FIGO (International Federation of Gynecology and Obstetrics) system and the AJCC (American Joint Committee on Cancer) TNM staging system. The pathological stage for OC was defined by three factors: the extent (size) of the tumor, spread to nearby lymph nodes and spread (metastasis) to distant sites [17]. FIGO stage was defined as stage I, II, III and IV [18]. Briefly: Stage I: Tumor limited to one or both ovaries, and fallopian tube and has not spread to nearby lymph nodes or organs within the pelvis or to distant sites; Stage II: Tumor is on the outer surface of or has grown into other nearby pelvic organs such as the uterus, bladder, the sigmoid colon, or the rectum and has not spread to nearby lymph nodes. Stage III: Tumor involves one or both ovaries with confirmed peritoneal metastases outside the pelvis and/or regional lymph node metastasis and Stage IV: Tumor had spread to distant organs (i.e. lung, liver) outside the abdominal region and/or distant lymph nodes. Note that the pathological staging is limited by the specimen submitted for pathological evaluation. If lymph nodes or tissues from distant sites are not submitted, the pathological stage will be dictated by the pathologist according to the available tissue. Final stage may be complemented with the information obtained through radiology and/or the clinical stage (patient’s symptoms, among other) ending in a late stage classification other than the one provided by pathologists. This represents a limitation of our study when evaluating tumor stage distribution in our sample.

BC histology was determined by a staff pathologist upon examination of H&E stained slides and was grouped into the following categories: invasive (included those described as “infiltrating”, “infiltrating duct” and “Invasive”), carcinoma in situ (included those described as “in situ” and “carcinoma *in situ*”) and invasive/in situ (those that were both invasive and in situ) [11]. Primary tumor size was determined by a pathologist by gross inspection and/or microscopically. We categorized continuous variable into 3 categories: less than 1 cm, 1–2 cm^3^, 2–5 cm^3^ and greater than 5cm^3^. Tumor grade was defined according to the American Joint Committee on Cancer [19] as follows: G1 (Well differentiated, low grade), G2 (Moderately differentiated, intermediate grade), G3 (Poorly differentiated, high grade), G4 (Undifferentiated, high grade). Primary tumor stage was defined according to the size and/or extent of the main tumor and skin ulceration following guidelines outlined by the American Joint Committee on Cancer [19].

#### Receptors status BC: Merging and subtype information

Expression levels of ER (estrogen receptor), PR (progesterone receptor), human epidermal growth factor receptor 2 (HER-2), Ki-67 and p53 were determined by IHC analyses following manufacturer’s protocols (See Additional file 1: Table S5 for information regarding antibody manufacturer and clone utilized in this study). Marker expression was analyzed by a pathologist through direct microscopic assessment or computer assisted evaluations (using the IScan Coreo AU system Virtuoso). ER, PR and HER-2, Ki-67 and p53 receptor status for each BC patient was identified by linking the information from the BrioQuery 6.6 database to the Breast Cancer Panel Reports. Databases were merged using the patient Case IDs. Data for BC receptor status information from the years 2000–2007 were not available. We classified BC cases using the criteria described by Millikan et al. [20]. In summary, subtype definitions were based upon three IHC markers: Luminal A (ER+ and/or PR+, HER-2–), Luminal B (ER+ and/or PR+, HER-2+), Triple negative (ER–, PR–, HER-2–), HER-2+ (ER–, PR–, HER-2+) and unclassified (cases without receptor status).

### Statistical methods

All statistics were performed using R version 3.2.4 [21]. Descriptive statistics were performed. Measures of spread and line graphs were performed on continuous variable age. Counts and percentages were performed on qualitative variables (cancer subtype, tumor size, tumor grade, tumor stage and histology). Tables were created for breast and ovarian cancer cases using counts and percentages. These tables were stratified by variables of interest (grade and cancer subtype). Trend lines were performed over time for the following: ovarian cancer cases with subtype and tumor grade, subtypes of ovarian cancer, subtypes of breast cancer, cancer grade evolution and caser cases with pathologic information. The distribution of cancer subtypes, total number of cancer cases, ovarian cancer cases, breast cancer cases, cancer grade were each graphed by 10-year age groups.

*For BC cases, s*ubtype evolution over time during the decade of life was also plotted as a percentage of both total cases and those cases with subtype information. Grade evolution over time was plotted and the coefficient of determination was calculated as a percentage of both total cases and those cases with grade information. Grade evolution over time was also plotted by decade of life. Total number of cases over time, along with pathologic information (subtype, grade, receptor information) was plotted over time for the total number of cases in this study. The total number of cases by age group was plotted.

*For OC cases,* the total number of cases, pathologic information (subtype, grade) were plotted over time for the total number of cases in this study. Subtype evolution over time and by age groups was also plotted, as was the distribution of total cases by age groups. Finally, grade evolution over time and by age groups was plotted.

## Results

### Ovarian Cancer

#### General characteristics

Data from 170 OC cases diagnosed between 2000 and 2015 were obtained from SPS records. Subtype information was available for 91.1% cases (*n* = 155; Table 1, Additional file 2: Figure S1A). Serous histology was identified in 64.7% (*n* = 110) of all cases and was consistently the most common subtype over time and across all age groups studied (Additional file 2: Figure S1B-C). Endometrioid histology was found in 10% (*n* = 17) of all cases, while 7.6% (*n* = 13) had mucinous and 2.3% (*n* = 4) clear cell histology. Mean age of diagnosis was 60.3 years (Table 1, Additional file 2: Figure S1D), Serous subtype having the highest age (61.4 years) and endometrioid having the lowest age (54.4 age) (Table 1; Additional file 3: Table S1). Most patients were from the South region of Puerto Rico (82.9%) (Table 1).

**Table 1:** Clinical characteristics of Ovarian Cancer cases by tumor subtype (2000–2015)

	Total *n* = 170	Clear/Squamous *n* = 4	Endometrioid *n* = 17	Mucinous *n* = 13	Serous *n* = 110	Other *n* = 11	Not spec *n* = 15
Overall % by subtypes	100%	2.35%	10%	7.65%	64.71%	6.47%	8.82%
Age Mean (SD) years	60.36 (12.64)	55.75 (20.41)	54.47 (11.47)	53.46 (11.34)	61.43 (12.17)	60.18 (15.05)	66.6 (11.04)
Location							
South	141 (82.94%)	4 (100%)	14 (82.35%)	11 (84.62%)	90 (81.82%)	9 (81.82%)	13 (86.67%)
West	28 (16.47%)	0	3 (17.65%)	2 (15.38%)	19 (17.27%)	2 (18.18%)	2 (13.33%)
North	1 (0.59%)	0	0	0	1 (0.91%)	0	0
Missing	–	–	–	–	–	–	–
Grade							
Other	2 (1.18%)	0	0	0	2 (1.82%)	0	0
G1	30 (17.65%)	1 (25%)	8 (47.06%)	7 (53.85%)	13 (11.82%)%)	0	1 (6.67%)
G2	33 (19.41%)	1 (25%)	7 (41.18%)	0	24 (21.82%)	1 (9.09%)	0
G3	82 (48.24%)	2 (50%)	1 (5.88%)	1 (7.69%)	66 (60%)	6 (54.55%)	6 (40%)
Missing	23	0	1	5	5	4	8
Stage							
Borderline	1 (0.59%)	0	0	0	1 (0.91%)	0	0
S1	61 (35.88%)	2 (50%)	13 (76.47%)	6 (46.15%)	31 (28.18%)	7 (63.64%)	2 (13.33%)
S2	25 (14.71)	0	1 (5.88%)	1 (7.69)	20 (18.18%)	0	3 (20%)
S3	59 (34.71)	0	1 (5.88%)	4 (30.77%)	44 (40%)	2 (18.18%)	8 (53.33%)
S4	8 (4.71%)	0	1 (5.88%)	0	5 (4.55%)	0	2 (13.33%)
Missing	16	2	1	2	9	2	0
Cancer type							
Invasive	9 (5.3%)	1 (25%)	1 (5.88%)	1 (7.69%)	5 (4.55%)	0	1 (6.67%)
Metastatic	86.0 (50.6%)	0	2 (11.76%)	5 (38.46%)	62 (56.36%)	4 (36.36%)	13 (86.67%)
Missing	75	3	14	7	43	7	1

One hundred and forty-seven (*n* = 147) cases had data on tumor grade (86.4%; Additional file 2: Figure S1A). Forty-eight percent (48.2%) were grade 3, 19.4% were grade 2, 17.6% were grade 1 and 1.18% of cases were borderline (Table 1). The most prevalent tumor grade was found to change over time with Grade 3 becoming more prevalent since 2007 in all age groups (Additional file 2: Figure S1E-F; Additional file 4: Table S2). Pathology reports showed that 90.5% (*n* = 86) of all cases in our cohort were metastatic, while the remaining 9.5% (*n* = 9) were invasive. This distribution was similar within the serous subtype (Table 1). Among cases with serous histology, the same trend was observed with tumor grade distribution. In our cohort, 153 ovarian cancer cases had tumor stage information (90.5%); *n* = 61 (35.8%) were stage 1, *n* = 25 (14.7%) were stage 2, *n* = 59 (34.7%) were stage 3 and *n* = 8 (4.7%) were stage 4. Serous subtype had a greater proportion of late stage (Stage 3/4) disease (*n* = 49, 44.5%).

#### IHC stains

Table 2 contains data regarding relevant biomarkers. Most tumors samples were not tested or data was not available to the researcher for these proteins (between 126 and 164 cases). Among the ones that were tested, most were P53 positive (overall = 72% and serous subtype = 81.2. %), ER positive (overall = 83.8% and serous subtype = 83.3%), CA125 positive (overall = 93.1% and serous subtype = 96.8%) and for Cytokeratin 5/6 (Overall = 55% and serous subtype = 80%). Staining for PR and Ki67 was distinct between both groups as most tumors were positive (71.4%) for PR, but the serous subtype showed a greater number of tumors that stained negative for this receptor (66.6%) and proliferation rates (Ki67 positivity) were moderate and high among serous subtype.

**Table 2:** Immunohistochemical analyses of relevant markers for Ovarian Cancer (2000–2015)

	Total *n* = 170	Serous *n* = 110
P53		
Negative	6 (3.53%)	3 (2.73%)
Positive	18 (10.59%)	13 (11.82%)
Borderline	1 (0.59%)	0
Missing	145	94
Estrogen Receptor		
Negative	5 (2.94%)	3 (2.73%)
Positive	26 (15.29%)	15 (13.64%)
Borderline	0	0
Missing	139	92
Progesterone Receptor		
Negative	4 (2.35%)	4 (3.64%)
Positive	10 (5.88%)	2 (1.82%)
Borderline	0	0
Missing	156	104
CA125		
Negative	1 (0.59%)	0
Positive	41 (24.12%)	31 (28.18%)
Borderline	2 (1.18%)	1 (0.91%)
Missing	126	78
Cytokeratin 5/6		
Negative	4 (2.35%)	1 (0.91%)
Positive	5 (2.94%)	4 (3.64%)
Borderline	0	0
Missing	161	105
Ki67 (Proliferative index)		
Low	0	0
Moderate	2 (1.18%)	2 (1.82%)
High	4 (2.35%)	2 (1.82%)
Missing	164	106

### Breast Cancer

#### General characteristics

**Fig. 1:**
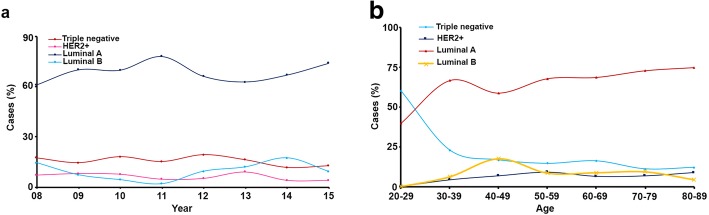
**a-b** Subtype evolution over time and age at diagnosis among breast cancer cases (2000–2015)

Data was obtained for 3451 BC cases from the SPS records between 2000 and 2015. Patients were mostly from southern (86.3%) Puerto Rico, reflecting the main area served by SPS. First, cases were divided into subtype categories; however, this information was missing from 2197 cases (63.7%; Table 3 and Additional file 5: Figure S2A). Out of samples with all receptor status available, the most common subtype was Luminal A (68.8%), followed by Triple negative (15.4%), Luminal B (9.7%), and HER-2+ (6.1%). Over the study time period, we observed no changes in overall prevalence of these subtypes; Luminal A remained the most common subtype, followed by Triple negative (Fig. 1a). The mean age of our cohort was 60.5 years (Additional file 5: Figure S2B), while Triple negative tumors had the youngest mean age of diagnosis (60 year) and Luminal A the oldest (63 years). Additionally, when patients were divided by decade of diagnosis, we observed that Triple negative cancers were the most commonly diagnosed subtype between the ages of 20–29 and declined over time. On the other hand, Luminal A diagnosis, steadily increased as patients became older (Fig. 1b and Additional file 6: Table S3).

**Table 3:** Clinical characteristics of Breast Cancer by tumor subtype (2000–2015)

	Total *n* = 3451	Luminal A *n* = 863	Luminal B *n* = 122	HER-2+ *n* = 76	Triple Neg *n* = 193	Unclassified *n* = 2197	Statistical test
Overall %	100%	25.00%	3.50%	2.20%	5.60%	63.70%	
% by subtype		68.80%	9.70%	6.10%	15.40%	–	
Age mean years (SD)	60.5 (12.8)	63 (12.4)	61 (12.4)	61 (12.8)	60 (13.5)	59 (12.8)	< 0.05^a^
Location							
Central	40 (1.2%)	–	–	–	–	40 (1.8%)	
East	26 (0.8%)	11 (1.27%)	4 (3.28)	–	5 (2.6%)	6 (0.3)	
North	11 (0.3%)	3 (0.4%)	–	–	3 (1.6%)	5 (0.2%)	
South	2979 (86.3%)	748 (86.7%)	108 (88.5%)	60 (79.0%)	154 (79.8%)	1909 (86.9%)	
West	395 (11.4%)	101 (11.7%)	10 (8.2%)	16 (21.1%)	31 (16.1%)	237 (10.8%)	
Missing	–	–	–	–	–	–	
Grade							< 0.001^b^
G1	297 (11.4%)	142 (20%)	8 (7.3%)	3 (4.9%)	4 (2.5%)	140 (9.0%)	
G2	1640 (63.1%)	452 (63.7%)	74 (67.3%)	36 (59.0%)	57 (35.0%)	1021 (65.7%)	
G3	660 (25.4%)	116 (16.3%)	28 (25.5%)	22 (36.1%)	102 (62.6%)	392 (25.2%)	
Missing	854	153	12	15	30	644	
Tumor size							< 0.05^b^
< 1 cm	500 (31.4%)	135 (36.2%)	15 (31.9%)	14 (36.8%)	18 (21.4%)	318 (30.2%)	
1-2 cm	468 (29.4%)	144 (38.6%)	15 (31.9%)	10 (26.3%)	30 (35.7%)	269 (25.6%)	
2-5 cm	518 (32.5%)	83 (22.3%)	15 (31.9%)	13 (34.2%)	31 (36.9%)	376 (35.7%)	
> 5 cm	108 (6.8%)	11 (2.9%)	2 (4.3%)	1 (2.6%)	5 (5.9%)	89 (8.5%)	
Missing	1857	490	75	38	109	1145	
Stage							< 0.001^b^
G1	102 (44.7%)	48 (53.3%)	2 (22.2%)	4 (26.7%)	11 (39.2%)	37 (43.0%)	
G2	102 (44.7%)	37 (41.1%)	6 (66.7%)	8 (53.3%)	11 (39.2%)	40 (46.5%)	
G3	23 (10.1%)	5 (5.6%)	1 (11.1%)	3 (20.0%)	5 (17.9%)	9 (10.5%)	
G4	1 (0.4%)	–	–	–	1 (3.6%)	–	
Missing	3223	773	113	61	165	2111	
Cancer type							< 0.001^b^
Invasive	319 (9.9%)	80 (9.5%)	6 (5.0%)	1 (1.4%)	8 (4.6%)	224 (11.1%)	
Carcinoma In-situ	769 (23.8%)	164 (19.6%)	31 (25.8%)	18 (24.7%)	20 (11.4%)	536 (26.5%)	
Invasive/In-situ	2131 (66.0%)	594 (70.9%)	83 (69.2%)	54 (74.0%)	147 (84.0%)	1253 (62.0%)	
Other	8 (0.2%)	–	–	–	–	8 (0.4%)	
Missing	224	25	2	3	18	176	

^a^Significance obtained from ANOVA test statistic, ^b^Significance obtained from Chi-Square test statistic

**Fig. 2:**
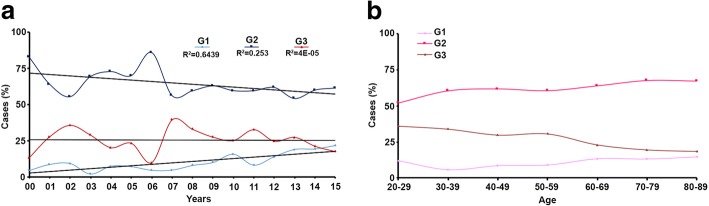
**a-b** Grade evolution over time and age at diagnosis among breast cancer cases (2000–2015)

Most BC cases had information on tumor grade (75.3%; Additional file 5: Figure S2A) and the distribution of tumor grades varied within different BC subtypes. The majority of Luminal A tumors were G2 (63.7%) with a small percent being G3 (16.3%). Whereas, most Triple negative tumors were G3 (62.6%) and very few G1 (2.5%). Over the period studied, we observed a decline in Grade 2 tumors and an increase in Grade 1 (Fig. 2a). When stratified by age at diagnosis, younger patients are diagnosed with more aggressive tumors when compared to older ones (Fig. 2b and Additional file 7: Table S4). Approximately one third of tumors (32.5%) measured between 2 cm to 5 cm, with Luminal A tumors being the smallest (74.8% measured 2 cm or less) and Triple negative tumors having the highest proportion of tumors measuring > 2 cm (42.8%). For 3223 cases, there was no information regarding cancer stage (93.4%). From the remaining 228 cases, 44.7% were diagnosed with stage 1, 44.7% were in stage 2, 10.1% were in stage 3, and 0.4% were in stage 4. Most BC tumors had both invasive and in situ components (66.0%).

#### IHC stains

Table 4 summarizes tumor staining information for clinically relevant biomarkers, including P53. Again, the vast majority of tumors were not tested or data was not available to the researcher for these markers. Our cohort had 1311 (38%) patients tested for P53. Among these, 72.2% were positive. The largest proportion of P53 positive tumors was among Luminal A (83.1%). In contrast, Triple negative tumors were P53 positive in 47.2% of all cases tested. Cytokeratin 5/6 tests were performed in 281 cases. Most tested negative (76.5%). Ki67 was performed in 983 samples and the about half were positive. However, when categorized by subtypes, 59.7% of Luminal A cases were positive for Ki67 whereas in the HER-2+ and Triple negative subtypes that percent was much lower (28.3 and 20.7% respectively).

**Table 4:** Immunohistochemical analyses of relevant markers for Breast Cancer (2000–2015)

	Total *n* = 3451	Luminal A *n* = 863	Luminal B *n* = 122	HER-2+ *n* = 76	Triple Neg *n* = 193	Unclassified *n* = 2197	Statistical test
P53							< 0.001^b^
Negative	363 (27.7%)	131 (16.9%)	38 (35.2%)	42 (61.8%)	93 (52.8%)	59 (31.9%)	
Positive	947 (72.2%)	643 (83.1%)	70 (64.8%)	26 (38.2%)	83 (47.2%)	125 (67.6%)	
Borderline	1 (0.0%)	–	–	–	–	1 (0.5%)	
Missing	2140	89	14	8	17	2012	
Cytokeratin 5/6							< 0.01^a^
Negative	215 (76.5%)	101 (70.6%)	32 (94.1%)	7 (77.8%)	25 (92.6%)	50 (73.5%)	
Positive	66 (23.5%)	42 (29.4%)	2 (5.9%)	2 (22.2%)	2 (7.4%)	18 (26.5%)	
Missing	3170	720	88	67	166	2129	
Ki67							< 0.001^b^
Negative	512 (52.1%)	222 (39.6%)	40 (60.6%)	37 (69.8%)	103 (76.3%)	110 (65.5%)	
Positive	459 (46.7%)	335 (59.7%)	26 (39.4%)	15 (28.3%)	28 (20.7%)	55 (32.7%)	
Borderline	12 (1.2%)	4 (0.7%)	–	1 (1.9%)	4 (3.0%)	3 (1.8%)	
Missing	2468	302	56	23	58	2029	

^a^Significance obtained from ANOVA test statistic ^b^Significance obtained from Chi-Square test statistic

## Discussion

In the present study, we conducted a retrospective analysis of pathology record data from breast and ovarian cancer patients between years 2000–2015 from a large pathology laboratory in the southwestern area of Puerto Rico. The data presented here highlights intriguing observations regarding breast and ovarian cancers that are specific to Hispanic patients from Puerto Rico. First, we observed a low prevalence of ovarian cancer cases in that time frame and their aggressive nature. Second, we identified intrinsic BC subtype distribution, their trends overtime and age at diagnosis. Finally, we noted that there is a significant amount of cases missing critical clinical and pathological information, especially in breast cancer. To the best of our knowledge, this study represents one of the most complete reviews of BC and OC pathological data from Puerto Rican patients. This study highlights the basic epidemiology of ovarian and breast cancer and suggests areas of health disparities in this population.

OC is typically diagnosed at advanced stages. Interestingly, there is no reliable screening biomarker and no recommended screening procedure for women in the general population [5, 22, 23]. Although transvaginal ultrasounds and serum CA-125 levels have been evaluated as screening methods for OC, they have not proven effective and do not improve survival rates [24]. In Puerto Rico, a study focused on OC screening practices among gynecologists/obstetricians in the island showed that 53.9% performed OC screening on asymptomatic patients, contrary to the American College of Obstetricians and Gynecologists and the Society of Gynecologic Oncology guidelines that do not recommend screening in low risk women [25]. Similar to our study, another investigation compared ethnic disparities among OC patients in the US and reported that the majority of the Hispanic cases (including Puerto Rican women) had a higher proportion of serous tumors and grade 3 classification [23]. Additionally, a population-based analysis of Hispanic women living in the US found that Hispanic women presented a higher proportion of cases that were diagnosed at earlier stages (I-II) [22]. This is similar to our findings that showed that the majority of the cases analyzed (50.51%) presented tumors at stage I and II.

Over the past years, BC incidence has remained stable among Hispanic women in the US [4]. However, it remains the most commonly diagnosed cancer and the leading cause of cancer-related deaths in this population [26]. Hispanic women in the US and in Puerto Rico have a lower risk of incident BC compared to non-Hispanic women [27]. However, they experience a slightly worse 5-year-survival after diagnosis [28]. BC is not a single disease, but rather a heterogeneous group of diseases that should be managed differently based on molecular and histological profiles. IHC assessment of ER, PR, and HER-2 expression breast cancers allows classification of tumors into distinct subtypes: that present distinct (1) etiologies, (2) incidence, (3) survival and (4) response to treatment [29–31].

Several studies have used IHC surrogates to classify breast tumors from patients living in the US (this population mostly represents Mexican-American women) [27]. In these studies, the range of incidence of different subtypes are as follows: Luminal A = 50.7–62.6%, Luminal B = 12.8–17.4%, HER-2 = 8.1–24.0% and Triple negative = 4.5–21.9% [32–36]. In San Juan, Puerto Rico, one study evaluated distribution of subtypes among a hospital based-cohort (*n* = 1072) where they found 61.8% of Luminal A patients, 13.3% Luminal B patients, 7.5% of HER-2 patients and 17.3% of Triple negative patients [14]. Another study focused on the clinicopathological factors associated with HER-2 status (*n* = 1049) in Puerto Rican patients, and reported a prevalence of 22.2% of HER-2 positivity, similar to that of the US [37]. Our results follow the expected subtype distribution (Luminal A = 68.8%, Luminal B = 9.7%, HER-2 = 6.1 and Triple negative = 15.4%), and these distributions remained stable over the time period studied. Slight discrepancies in incidence with other Puerto Rico studies may arise due to sociodemographic differences in our study population. For example, the study conducted in San Juan is mostly a metropolitan based cohort versus our rural based cohort. Other differences might be due to the period in which the studies were conducted. The most common diagnosed tumors were grade 2, as previously reported by Ortiz et al. [37]. On the other hand, trends for grades change over the time period studied. Grade 2 tumors declined over time whereas grade 1 tumors increased. This is consistent with previously presented data suggesting that these differences may be the result of increased educational efforts across the island in recent years and the implementation of mammography screening techniques [38]. Moreover, the Healthy People 2020 initiative main target is to reduce late-stage BC diagnoses to 38.9 per 100,000 patients and we believe that this downward trend should continue in the foreseeable future [39]. The mean age of BC diagnosis in our study cohort was 60.5 years, which is comparable to the national US average [2] and the mean age reported by the National Cancer Registry of Puerto Rico [38]. We observed that more aggressive tumors and higher grades were diagnosed at earlier ages, similar to previously reported trends for African American populations in the US [20]. This observation supports the findings from a recent study demonstrating that Puerto Rican women with higher proportions of African ancestry are at increased risk for Triple negative and more aggressive tumors [40].

In addition to analyzing ovarian and breast cancer patient characteristics, we also describe possible ‘gaps’ in-patient care that could underlie outcome disparities. Our study shows that some OC cases did not receive either a histological subtype diagnosis, grade, stage or histological markers in their pathology report. Recent efforts are aimed at understanding and determining the characteristic of each subtype of ovarian tumors [39]. Because ovarian tumors exhibit histological heterogeneity and each subtype display different cellular and morphological characteristics, histological knowledge of these OC tumors is important to optimize treatment options for this disease and reduce mortality of OC patients. Therefore, it is critical to have accurate and complete knowledge of tumor sample characterization that could help researchers identify personalized treatment options that could ultimately improve ovarian cancer patient prognosis.

We also observed significant gaps in the documentation of hormone receptor expression in BC patients. This may be due to the fact that it was not until 2008 when ER, PR and HER-2 analyzes became common practice across pathology laboratories in the island and were included in electronic records. Thus, we expected that from 2008 onward this information would be recorded; however, it was missing for a high amount of cases (Additional file 5: Figure S2). This may be a consequence of several factors, such as 1) Study limitations (i.e. limited access by the researcher to all the pathological information of the same patient contained in multiple sources or reports, such as HER-2 status determined by FISH rather than by IHC as only IHC data was evaluated in this study); 2. Health insurance coverage limitations (i.e. the test was not performed because the health insurance did not covered either the IHC test or the HER-2 reflex test by FISH for those cases with an initial HER-2 equivocal (2+) result by IHC); 3. Information of the tests performed on the tissue resection (after the initial diagnosis performed in a biopsy) was missing because for the purpose of this study only the initial diagnosis report was used and all the secondary reports were excluded from the analysis. Because BC standard of care is based on the tumor characteristics [41], lack of these data could lead to sub-optimal treatment plans and lead to outcome disparities. The results of our analyses suggest that many women with BC might not be receiving the appropriate management for their disease and in some cases, may be undertreated. On the other hand, some may be over-treated leading to unnecessary personal and social stress [42, 43]. The fact that the ‘unclassified’ group presents the youngest mean age (59.2 years) and highest proportion of larger tumors (> 5 cm) may indicate a bias towards more aggressive cancers not being treated adequately. Future, studies should be conducted to address these knowledge gaps.

The main limitation of this study lies on its dependence in data captured from pathology reports. Therefore, important demographical, risk factor and care information were not available. Additionally, follow-up information for these patients was not possible given the blinded nature of the study. These factors limit the scope of this study and do not allow our group to perform more complex and necessary analyses. The fact that many reports are missing critical tumor information also reduce the capacity to draw relevant conclusions about tumor and patient characteristics in this population. Future efforts should focus on understanding tumor etiology through comprehensive analysis of known risk factors, as well as carcinogenesis with essential information on treatment and patient follow-up in Puerto Rico. Despite these limitations, this study is important as it focuses on Hispanic women living in Puerto Rico and provides the opportunity to study this population separate from other US Hispanic populations. Most epidemiological studies conducted thus far consider Hispanic women in the USA as one homogenous group. However, this ethnic community is one of the most diverse in terms of origin and culture and provides both challenges and opportunities to study carcinogenesis and cancer etiology. Additionally, given the lack of up-to-date Puerto Rico-wide epidemiological studies, the work presented here provides a historical perspective from the past 15 years in Puerto Rico. Based on data from the National Cancer Registry of Puerto Rico, our study captures 15% of the total BC cases and 9.6% of all OC cases in PR during the 2000–2015 period [38]. Lastly, this study is one of the very few to have evaluated breast and ovarian cancer subtype distribution among Hispanic women from Puerto Rico. The documented high prevalence of cases where subtype or other clinical data was missing could be the result of study limitations and/or a health disparity that leads to worse diagnosis, treatment and disease outcome in this geographical area that needs to be addressed as soon as possible.

## Conclusions

In conclusion, with Puerto Rico missing from US nation-wide studies it is difficult to perform an up-to-date assessment on health disparities on the island of Puerto Rico. Our data suggests that OC and BC subtypes distribution in Hispanic populations from PR are comparable to reported national averages. Moreover, a significant number of cases subtypes, especially for BC, could not be determined as a consequence of study limitations, health insurance coverage, or other reasons described here and may constitute a health disparity. Studies similar to this one can start to shed light on the specific characteristics of BC and OC patients in Puerto Rico (such as comprehensive follow-up data based on BC and OC subtypes) and highlight knowledge gaps that can be exploited to develop better treatment options for these patients.

## Additional files

Additional file 1: **Table S5.** Manufacturer and clone information for antibodies used for IHC analyses. (DOCX 18 kb)

Additional file 2: **Figure S1.** Description of Ovarian Cancer cases included in this study. **a)** Total number of Ovarian Cancer cases over time and pathologic information. **b)** Ovarian Cancer subtype evolution over time. **c)** Ovarian Cancer subtypes by age group. **d)** Total number of ovarian cancer cases by age group. **e)** Ovarian Cancer grade evolution over time. **f)** Ovarian Cancer grade by age group. (JPG 856 kb)

Additional file 3: **Table S1.** Total number of Ovarian Cancer cases by age group and subtype. (DOCX 14 kb)

Additional file 4: **Table S2.** Total number of Ovarian Cancer cases by age group and grade. (DOCX 13 kb)

Additional file 5: **Figure S2**. Description of Breast Cancer cases included in this study. **a)** Total number of Breast Cancer cases over time and pathologic data. **b)** Total number of Breast Cancer cases by age group. (JPG 330 kb)

Additional file 6: **Table S3.** Total number of Breast Cancer cases by age group and subtype. (DOCX 13 kb)

Additional file 7: **Table S4.** Total Number of Breast Cancer cases by age group and grade. (DOCX 13 kb)

## Abbreviations

BC: Breast Cancer
ER: Estrogen Receptor
H&E: Hematoxylin and Eosin
HER-2: Human Epidermal Growth Factor 2
IHC: Immunohistochemistry
OC: Ovarian Cancer
PR: Progesterone Receptor
