# Supplementary material for: Prevalence of breast and ovarian cancer subtypes in Hispanic populations from Puerto Rico
Source: BMC Cancer. 2018 Nov 27;18:1177. doi: 10.1186/s12885-018-5077-z (PMC6260719; doi:10.1186/s12885-018-5077-z)
Supplement: Supplementary file 1 — Table S5. Manufacturer and clone information for antibodies used for IHC analyses. (DOCX 18 kb) [file 12885_2018_5077_MOESM1_ESM.docx]

Table S5. Manufacturer and clone information for antibodies used for IHC analyses

| Marker | Clone | Company |
| --- | --- | --- |
| P53 | D0-7 | VENTANA DAKO |
| Ki67 | 30-9 | VENTANA |
| Ki67 | MIB-1 | DAKO |
| ESTROGEN | EP1 | DAKO |
| ESTROGEN | IE2 | VENTANA |
| PROGESTERONE | PgR 636 | DAKO |
| PROGESTERONE | SP1 | VENTANA |
| CD10 | 56C6 | DAKO |
| CA125 | M11 | DAKO |
| HER-2 | 4B5 | VENTANA |
