# Supplementary figures and images for: Prevalence of breast and ovarian cancer subtypes in Hispanic populations from Puerto Rico
Source: BMC Cancer. 2018 Nov 27;18:1177. doi: 10.1186/s12885-018-5077-z (PMC6260719; doi:10.1186/s12885-018-5077-z)

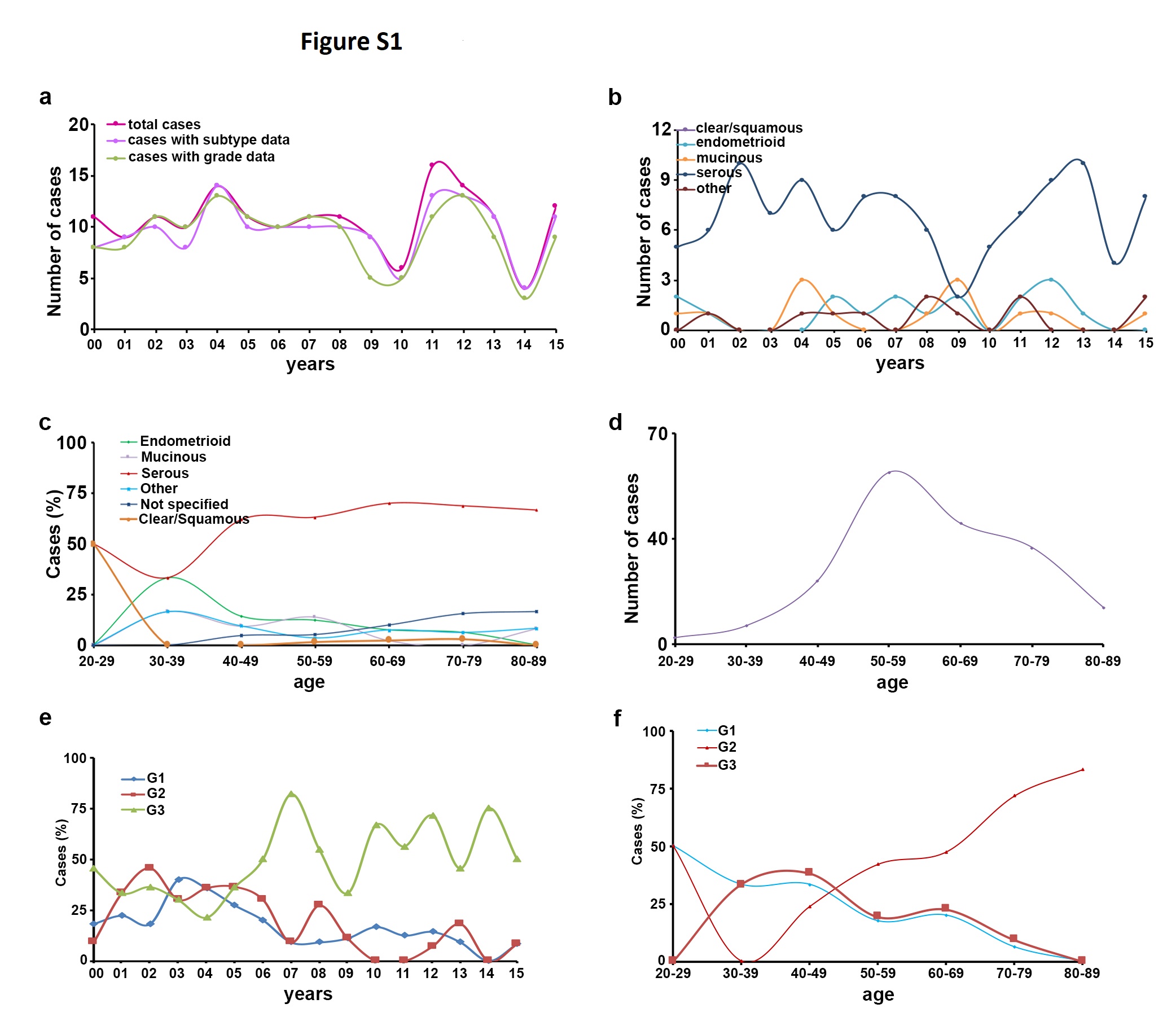

Supplement: Supplementary file 2 — Figure S1. Description of Ovarian Cancer cases included in this study. a) Total number of Ovarian Cancer cases over time and pathologic information. b) Ovarian Cancer subtype evolution over time. c) Ovarian Cancer subtypes by age group. d) Total number of ovarian cancer cases by age group. e) Ovarian Cancer grade evolution over time. f) Ovarian Cancer grade by age group. (JPG 856 kb) [file 12885_2018_5077_MOESM2_ESM.jpg]

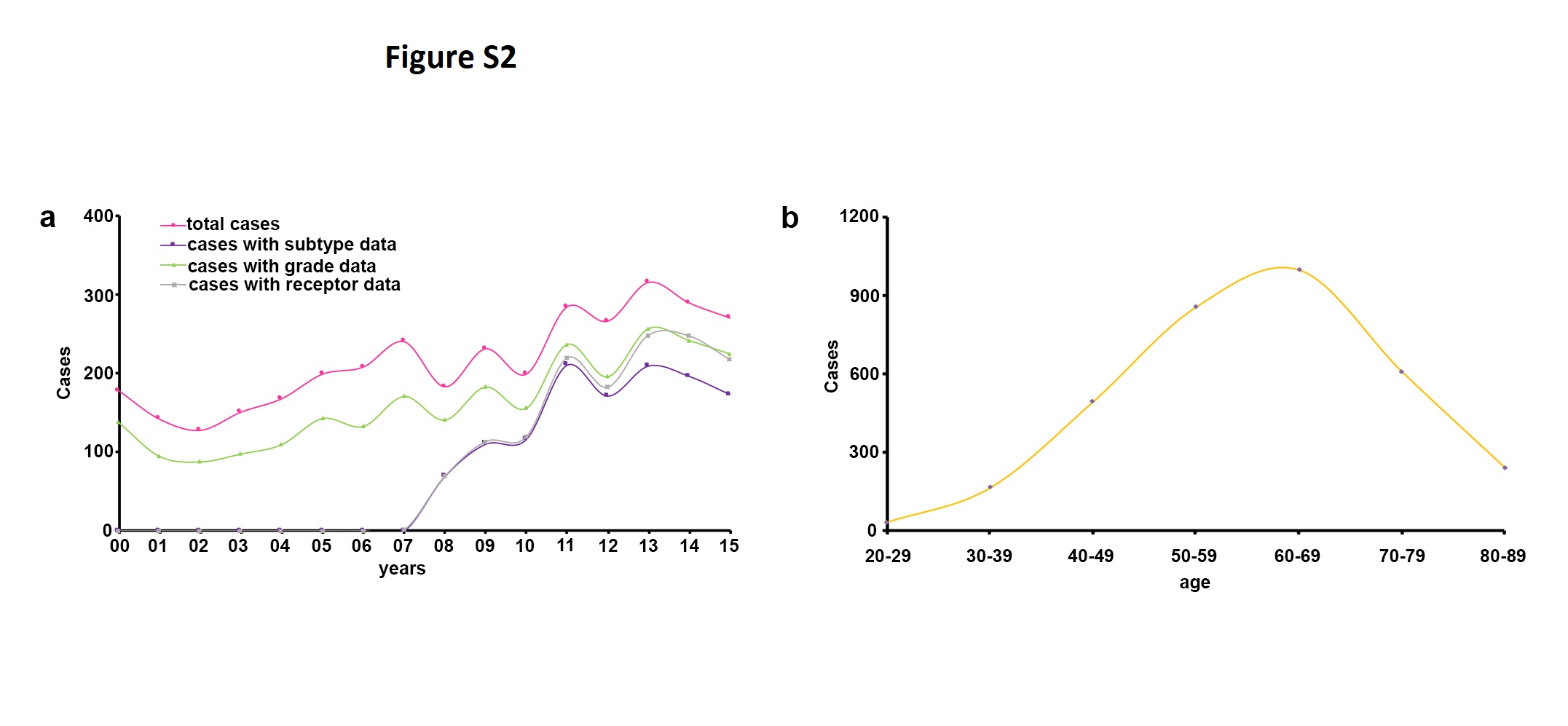

Supplement: Supplementary file 5 — Figure S2. Description of Breast Cancer cases included in this study. a) Total number of Breast Cancer cases over time and pathologic data. b) Total number of Breast Cancer cases by age group. (JPG 330 kb) [file 12885_2018_5077_MOESM5_ESM.jpg]
