# Supplementary material for: Prevalence of breast and ovarian cancer subtypes in Hispanic populations from Puerto Rico
Source: BMC Cancer. 2018 Nov 27;18:1177. doi: 10.1186/s12885-018-5077-z (PMC6260719; doi:10.1186/s12885-018-5077-z)
Supplement: Supplementary file 3 — Table S1. Total number of Ovarian Cancer cases by age group and subtype. (DOCX 14 kb) [file 12885_2018_5077_MOESM3_ESM.docx]

Table S1. Total number of Ovarian Cancer cases by age group and subtype

| Age Group (years) | Clear/Squamous | Endometrioid | Mucinous | Serous | Other | Not specified | Total |
| --- | --- | --- | --- | --- | --- | --- | --- |
| 20-29 | 1 | 0 | 0 | 1 | 0 | 0 | 2 |
| 30-39 | 0 | 2 | 1 | 2 | 1 | 0 | 6 |
| 40-49 | 0 | 3 | 2 | 13 | 2 | 1 | 21 |
| 50-59 | 1 | 7 | 8 | 36 | 2 | 3 | 57 |
| 60-69 | 1 | 3 | 1 | 28 | 3 | 4 | 40 |
| 70-79 | 1 | 2 | 0 | 22 | 2 | 5 | 32 |
| 80-89 | 0 | 0 | 1 | 8 | 1 | 2 | 12 |
