# Supplementary material for: Prevalence of breast and ovarian cancer subtypes in Hispanic populations from Puerto Rico
Source: BMC Cancer. 2018 Nov 27;18:1177. doi: 10.1186/s12885-018-5077-z (PMC6260719; doi:10.1186/s12885-018-5077-z)
Supplement: Supplementary file 6 — Table S3. Total number of Breast Cancer cases by age group and subtype. (DOCX 13 kb) [file 12885_2018_5077_MOESM6_ESM.docx]

Additional file Table 3. Total number of Breast Cancer cases by age group and subtype

| Age Group (years) | Luminal A | Luminal B | HER-2+ | Triple Negative | TOTAL |
| --- | --- | --- | --- | --- | --- |
| 20-29 | 3 | - | - | 4 | 7 |
| 30-39 | 34 | 3 | 3 | 13 | 53 |
| 40-49 | 85 | 24 | 11 | 28 | 148 |
| 50-59 | 181 | 25 | 20 | 39 | 265 |
| 60-69 | 265 | 35 | 20 | 61 | 381 |
| 70-79 | 186 | 26 | 11 | 30 | 253 |
| 80-89 | 82 | 6 | 7 | 12 | 107 |
