# Supplementary material for: Prevalence of breast and ovarian cancer subtypes in Hispanic populations from Puerto Rico
Source: BMC Cancer. 2018 Nov 27;18:1177. doi: 10.1186/s12885-018-5077-z (PMC6260719; doi:10.1186/s12885-018-5077-z)
Supplement: Supplementary file 7 — Table S4. Total Number of Breast Cancer cases by age group and grade. (DOCX 13 kb) [file 12885_2018_5077_MOESM7_ESM.docx]

Additional file Table 4. Total Number of Breast Cancer cases by age group and grade

| Age Group (years) | Grade 1 | Grade 2 | Grade 3 | TOTAL |
| --- | --- | --- | --- | --- |
| 20-29 | 3 | 13 | 9 | 25 |
| 30-39 | 7 | 73 | 41 | 121 |
| 40-49 | 32 | 230 | 111 | 373 |
| 50-59 | 57 | 382 | 193 | 632 |
| 60-69 | 100 | 477 | 171 | 748 |
| 70-79 | 60 | 306 | 88 | 454 |
| 80-89 | 28 | 128 | 35 | 191 |
